# Clinical Application of Myocardial Perfusion SPECT in Patients with Suspected or Known Coronary Artery Disease. What Role in the Multimodality Imaging Era? 

**DOI:** 10.31083/j.rcm2402048

**Published:** 2023-02-06

**Authors:** Caterina Maffeis, Francesco Dondi, Flavio Luciano Ribichini, Raffaele Giubbini, Alessia Gimelli

**Affiliations:** ^1^Cardiology Division, Department of Medicine, University of Verona, 37126 Verona, Italy; ^2^Nuclear Medicine, ASST Spedali Civili Brescia, 25123 Brescia, Italy; ^3^Department of Nuclear Medicine, University of Brescia, 25123 Brescia, Italy; ^4^Cardiovascular and Imaging Departments, CNR Research Area, Fondazione CNR/Regione Toscana Gabriele Monasterio, 56124 Pisa, Italy

**Keywords:** nuclear imaging, myocardial perfusion scintigraphy, coronary artery disease, multimodality imaging

## Abstract

Myocardial perfusion single photon emission computed tomography (SPECT) is widely used in assessing coronary artery disease (CAD) owing to its proven efficacy in extensive clinical experience. Like other functional tests, myocardial SPECT is recommended for the diagnosis of obstructive CAD, risk stratification assessment, and treatment decision making. Besides quantifying left ventricular volume, global and regional function by electrocardiography (ECG)-gated acquisition, myocardial SPECT can identify myocardial ischemia, scars, stunning, and viable hibernating myocardium. It provides comprehensive functional data across the spectrum of CAD and a cost-effective strategy in patients with intermediate pre-test probability of CAD or with a history of ischemic cardiomyopathy. With ongoing advances in cardiovascular prevention and risk factor management many patients referred for testing now have a low-to-intermediate probability of CAD. Besides, CAD has become a chronic condition resulting from novel therapeutic strategies. Against this background, approaches combining anatomical and functional tests in sequence or simultaneously include coronary artery calcium score integrated with perfusion imaging or fusion SPECT/coronary computed tomography angiography (CCTA). In this review we summarize current indications for myocardial perfusion SPECT and integration of SPECT with other imaging techniques to improve diagnostic performance, patient management, and outcome prediction in CAD.

## 1. Introduction

Myocardial perfusion imaging (MPI) constitutes a milestone in diagnosis and management of coronary artery disease (CAD). By virtue of its ability to detect stress-induced myocardial perfusion defects, single photon emission computed tomography (SPECT) is a primary tool for diagnosis of obstructive CAD. It is also useful in risk stratification of patients with suspected or known CAD [1, 2, 3]. Finally, functional SPECT data on total ischemic burden, ischemia site, extension and severity can inform treatment decisions [4].

The shared view of the “ischemic issue” was embodied by the ischemic cascade [5] and the principle that greater coronary stenoses trigger more severe ischemia. Perfusion abnormalities were thought to occur soon in the ischemic cascade and derive from stenoses of borderline significance, giving reason for the high sensitivity of SPECT in detecting obstructive CAD. Furthermore, the degree and the extent of ischemia was assumed to be directly related to event risk. Later evidence showed, however, that, because of the effect of atherosclerotic plaque and coronary microvasculature features on myocardial perfusion, interaction between atherosclerosis, stenosis, and ischemia is not linear [6]. In addition, the prognostic implications of anatomical and functional abnormalities vary widely. The results from trials like FAME (Fractional Flow Reserve versus Angiography for Multivessel Evaluation) [7], ORBITA (Objective Randomized Blinded Investigation with Optimal Medical Therapy of Angioplasty in Stable Angina) [8], and ISCHEMIA (International Study of Comparative Health Effectiveness with Medical and Invasive Approaches) [9] have changed our perspective on myocardial ischemia and its implications for prognosis and clinical decision-making.

With the present review we summarize indications for myocardial perfusion SPECT in patients with suspected or known CAD and potential applications of MPI in multimodality imaging.

## 2. Pathophysiologic Basics and Rationale for Myocardial Perfusion SPECT

Since cardiac metabolism is predominantly aerobic, it depends on continuous extraction of oxygen from coronary blood flow. Normal coronary flow to the left ventricle under basal conditions is 0.6–1 mL/min/g but it can increase up to six fold during increased demand. Cardiac arterioles and capillaries control blood flow according to metabolic needs and are the main determinants of coronary flow resistance. In detail, small coronary arteries (<500 μm) can regulate myocardial perfusion and blood flow [10]. In this scenario, coronary flow reserve refers to the ability to raise flow rate from a basal level at rest to a maximal level during exercise [11]. Atherosclerosis can reduce such reserve, however. When a coronary artery is narrowed by more than 50%, blood flow cannot increase sufficiently during stress. This produces a mismatch between blood supply and oxygen demand, ensuing in myocardial ischemia [12]. Also, when blood supply is reduced, myocardial contraction is reduced or stops, leading to a regional-wall-motion abnormality during ischemia. In this setting, when the area supplied by the stenotic coronary artery is extensive, left ventricular function may be impaired and ejection fraction reduced.

In the diagnosis of ischemia, there are two techniques to detect stenosis of the epicardial coronary arteries by increasing coronary flow: exercise or pharmacological stressors that increase oxygen consumption or flow nonuniformity through vasodilatation [12]. The physiological method to increase coronary flow is physical effort. Pharmacological agents (mainly the vasodilators dipyridamole, adenosine, and regadenoson and the sympathomimetic dobutamine) can be used in patients unable to adequately exercise. In this setting, MPI with SPECT are applied to evaluate coronary blood flow in stress and rest conditions by means of radioactive tracers ([^201^Tl]TlCl, ^99m^technetium [^99m^Tc] labeled with sestamibi or tetrofosmin), which are extracted by the cardiomyocytes and trapped in the mitochondria. While myocardial tracer uptake is flow-dependent, the relation between coronary flow and heart uptake is non-linear, especially at high flow rates [13, 14, 15]. Myocardial retention of these radiopharmaceuticals is relatively long, compatible with SPECT acquisition time, whereas clearance from circulation is rapid. This difference allows the distribution of coronary blood flow to the myocardium to be mapped. Two tracer injections to assess stress and resting perfusion are generally given on different days or the same day according to protocol (dual-day or single-day protocol). If the stress test is performed first and perfusion results normal, the rest examination can be omitted (stress-only protocol) [16], whereas when flow distribution into the myocardium is not uniform, the myocardial area where the tracer retention is relatively lower identifies the region with decreased perfusion, and a rest test is indicated. A regional perfusion defect after stress but absent at rest identifies myocardial ischemia and suggests the anatomical site of coronary stenosis and its extension and severity [17]. A region with reduced perfusion after stress and at rest is defined as a myocardial scar (Table 1).

**Table 1.** **Left ventricular myocardial states according to SPECT findings**.

Myocardial state	Description	Perfusion SPECT	Gated SPECT
Normal	Normal myocardial perfusion and function	Normal stress and rest perfusion	Normal function
Ischemia	Reversible perfusion defect with or without myocardial functional abnormalities due to transient reduction in coronary blood flow	Reduced stress and normal rest perfusion	Normal or reduced during stress
Stunned myocardium	Myocardial dysfunction during stress which persists at rest despite restored normal resting myocardial blood flow; it can occur after stress-induced reversible ischemia or in myocardial infarction after restoration of coronary flow in a previously occluded vessel; myocardial function recovers after a variable amount of time	Reduced stress and normal rest perfusion	Reduced during and after stress, improved at rest
Hibernating myocardium	Dysfunctional but viable myocardium with mildly reduced resting myocardial blood flow resulting from repetitive ischemia and leading to chronic myocardial ischemia	Reduced stress and rest perfusion	Reduced function
Scar	Severely dysfunctional and not viable myocardium with severely reduced resting myocardial blood flow	Severely reduced stress and rest perfusion	Reduced function

**Fig. 1.**
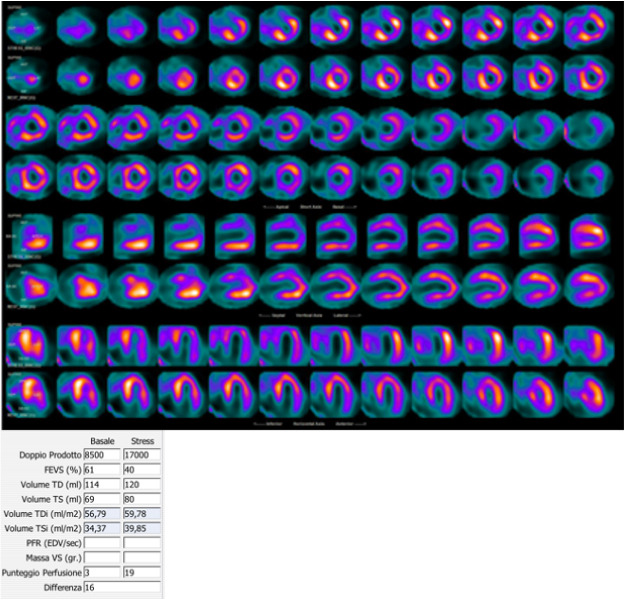
**99mTc-tetrofosmin cardiac SPECT with cadmium, zinc, tellurium (CZT) technology images of a 72-year-old woman with multiple cardiovascular risk factors symptomatic for typical effort angina**. Perfusion SPECT revealed a reversible defect in the apex, septal, and anterior walls. Functional data showed an increase in left ventricular end-diastolic volume and end-systolic volume after stress compared to rest (120 versus 114 mL and 80 versus 69 mL, respectively); global systolic function was reduced after stress normal at rest (ejection fraction 40 versus 61%), compatible with myocardial stunning.

**Fig. 2.**
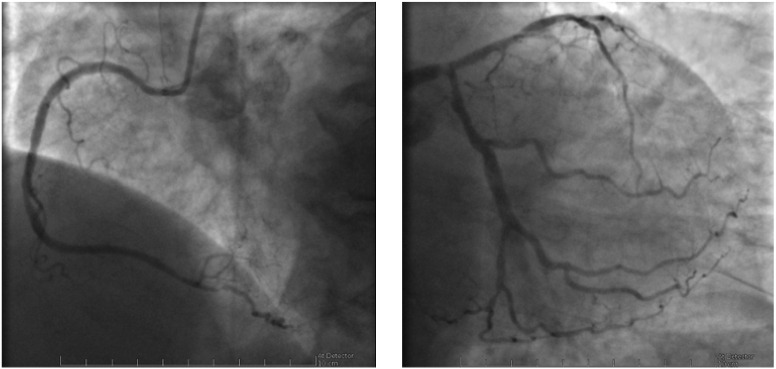
**Coronary angiography images of the case described in Fig. 1 showing diffuse CAD, with severe stenosis of the left main artery**. Left: right coronary artery; Right: left main, anterior descendent and circumflex arteries.

Reduced blood flow to the myocardium during stress and subsequent ischemia can impair left ventricular contractility and regional or global function eventually. One of the advantages of cardiac SPECT is its utility in functional assessment via electrocardiography (ECG)-gated acquisition. Left ventricular ejection fraction, volume, mass, myocardial systolic wall thickening, and contraction dyssynchrony can be quantified [18, 19], thus providing detailed information beyond those characteristic of myocardial perfusion imaging. ECG-gated acquisition in the stress phase of SPECT can detect myocardial stunning [6, 20]. During ischemia, a regional perfusion deficit may be associated with impaired contractility in the same region. After blood flow is restored, abnormal cardiac function can persist for hours to days despite restoration of normal perfusion. Since SPECT acquisition occurs at least 15 minutes after radiopharmaceutical injection as per protocol, evidence of stress-induced regional perfusion impairment associated with a persistent mild alteration in motion or thickening in the same region is suggestive of stunned myocardium (Figs. 1,2). Evidence of stunning in >5% of the total myocardium is a predictor of adverse events [21, 22].

The assessment of MPI at rest is essential for evaluating cardiac viability and for differentiating impaired retention of perfusion tracers found in myocardial stunning from decreased uptake due to decreased perfusion in hibernation or scar [23, 24]. In patients with known CAD and prior infarction, the SPECT protocol includes tracer injection after nitrate infusion to improve detection of the viable hibernating myocardium [25]. The acute administration of nitrate increases radiopharmaceutical uptake in dysfunctional territories which show functional recovery after revascularization [26]. Furthermore, the combination of metabolic and perfusion imaging via ^18^F-fluorodeoxyglucose (^18^F-FDG) positron emission tomography/computed tomography can further differentiate between stunned and hibernated myocardium and scar.

## 3. Clinical Indications for Myocardial Perfusion SPECT: Guideline-Directed Testing

Coronary artery disease is the leading cause of death worldwide and a major public health problem. The age-adjusted prevalence of CAD is approximately 7% in males and 4% in females [27]; the prevalence is 25% and 16%, respectively, in adults aged 60 to 79 years and is even higher in those 80 years or older [28]. Early detection of CAD is a primary strategy to reduce the burden of cardiovascular disease and mortality. As a perfusion imaging technique, myocardial SPECT provides a non-invasive tool for diagnosing obstructive CAD in symptomatic or asymptomatic patients at intermediate or high cardiovascular risk. Clinical referral for SPECT has increased in Western countries [29] and myocardial SPECT is contemplated by guidelines for the management of patients with known or suspected CAD, including risk stratification, treatment decision making, and prognosis.

There is international consensus on performing myocardial SPECT in at least four clinical scenarios: suspected or known stable CAD, suspected acute coronary syndrome, before non-cardiac surgery, and heart failure.

### 3.1 Stable Coronary Artery Disease

American and European guidelines recommend the use of myocardial perfusion SPECT as a diagnostic tool in chronic chest pain and suspected or known CAD [30, 31]. Besides its use in diagnosis, SPECT provides prognostic information and can guide patient management.

#### 3.1.1 Suspected CAD

In patients with suspected CAD, non-invasive imaging modalities are the preferred method for diagnosing CAD [30], except in those with a high pretest probability in which coronary angiography is indicated instead (Class of Recommendation [COR] I, Level of Evidence [LOE] B [30], COR IIa, LOE C [32]) [31]. The application of myocardial SPECT or stress echocardiography is recommended in symptomatic patients with intermediate pre-test probability (PTP) of disease (arbitrarily defined between 15% and 85% estimated according to age, sex, and nature of symptoms [33]), especially in patients unable to exercise and with abnormalities at resting electrocardiography (COR I, LOE B). In patients with an intermediate PTP and an interpretable ECG and can adequately exercise (5 metabolic equivalents (METs) or more), exercise ECG may be considered an alternative according to the 2021 American guidelines (COR IIa, LOE B) [31]. However, the diagnostic utility of exercise ECG is lower than that of imaging testing. Most recent European guidelines [30] suggest that exercise electrocardiography be performed as a diagnostic tool only when other imaging modalities are unavailable (COR IIb, LOE B).

Non-invasive functional imaging to detect ischemia and anatomical imaging by coronary CT angiography (CCTA) are recommended in symptomatic patients. SPECT, because of its high rule-in power, is the preferred technique in patients at intermediate-high risk. SPECT has a diagnostic sensitivity of 82–88% for exercise and 88–91% for pharmacological stress testing and a specificity of 70–88% and 75–90%, respectively [5, 34]. Initial evaluation by stress MPI resulted in less downstream non-invasive and invasive testing in decision-making processes. In one study cohort [35], initial SPECT findings were abnormal in 29% of patients compared to 56% in those undergoing CCTA, and a high proportion of patients underwent a functional test following positive CCTA. Symptomatic patients with suspected CAD but normal SPECT findings have a favorable prognosis (0.6% event rate according to pooled analyses), similar to the general population risk [36]. Differently, abnormal findings may be suggestive of an increased event risk, in which the event rate is related to patient risk profile, left ventricular function, and ischemic burden [37].

Non-invasive tests may also be considered in patients with a PTP between 5% and 15%, especially when the clinical likelihood is greater due to risk modifiers such as cardiovascular risk factors, electrocardiographic abnormalities, left ventricular dysfunction, and coronary calcifications on CT findings [38, 39, 40, 41]. Data from the PROMISE trial showed a prevalence of CAD in 7% of patients with low CAD probability (<10%) based on a clinical model (including age, sex, symptoms, diabetes, hypertension, hyperlipidemia, smoking), whereas the prevalence was 2% in patients judged as having a low probability when the coronary artery calcium (CAC) score was included in the evaluation [42, 43]. These extended prediction models plus clinical and anatomical factors help clinicians to manage this low PTP population [31], identify patients with minimal-risk in which testing can be deferred [44], and order non-invasive diagnostic testing in patients with multiple risk features.

#### 3.1.2 Patients with Known CAD

**Fig. 3.**
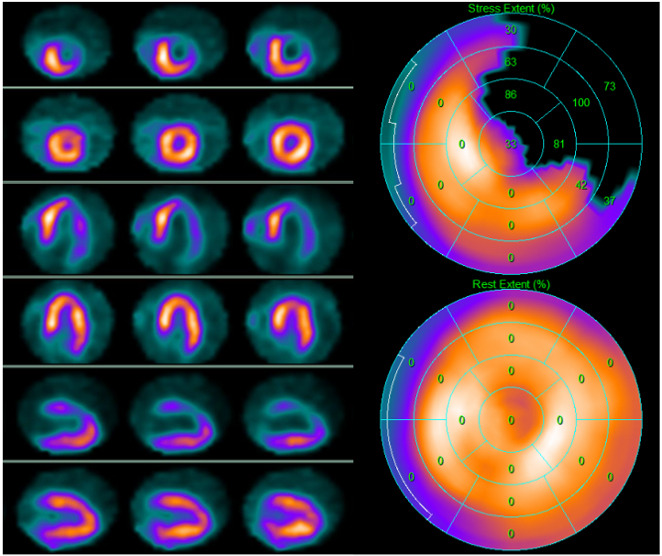
**99mTc-tetrofosmin cardiac single photon emission computed tomography (SPECT) images in a 74-year-old woman with arterial hypertension, diabetes mellitus, and a history of obstructive CAD without myocardial infarction treated with triple coronary artery bypass surgery five years earlier**. Perfusion imaging was ordered to assess ischemia due to persistent atypical angina after medical therapy optimization. SPECT revealed a large reversible defect in the anterior and the lateral walls showing severe myocardial ischemia. Subsequent angiography revealed complete occlusion of the left main artery, occlusion of the saphenous vein graft anastomosed to the diagonal branch of the left anterior descendent coronary artery, and patency of the other two bypass grafts. Medical therapy was optimized. Left side top row: stress-rest short axis; stress-rest horizontal long axis; stress-rest vertical long axis; right side: polar map of stress (upper image) and rest perfusion (lower image).

In patients with known CAD, the utility of myocardial SPECT relies on its ability to define the site and severity of ischemia. In this population, guidelines-directed medical therapy is key to slowing disease progression, improving symptoms, and preventing acute atherothrombotic events, as well as relief from angina and prognosis improvement. In patients with stable chest pain, guidelines currently recommend an ischemia-based strategy to guide revascularization, with the use of stress imaging to estimate disease severity and identify target lesion(s), especially in moderate coronary stenoses at angiography and in lesions of uncertain functional significance. Applied in stress testing, myocardial SPECT can help identify the site and the extent of inducible perfusion defects, and the anatomical distribution of a hemodynamically significant coronary stenosis (Fig. 3).

Revascularization is indicated when large areas of ischemia are detected (>10% of the myocardium) (COR I, LOE B) [30]. Observational data of 43,443 patients undergoing perfusion SPECT demonstrated a survival advantage with early revascularization when the left ventricular ejection fraction (LVEF) was normal (≥45%) and severe inducible ischemia was detected (≥15% total myocardium), but also, to a less extent, when the LVEF was low (<45%) and inducible ischemia was moderate or severe (>10% total myocardial ischemia). In contrast, an increased mortality risk with revascularization was demonstrated in the absence of ischemia [1], further confirming previous published studies [45, 46, 47]. In addition to ischemia severity, the detection of high-risk imaging features like transient ischemic ventricular dilation is of prognostic relevance. The REFINE SPECT registry (involving 16,578 patients) reported that the outcome of those presenting with mild ischemia and transient ischemic dilation or post-stress wall motion abnormalities was similar to those with moderate ischemia [3]. Furthermore, data from the recently published Analysis of Myocardial Ischemia by Cadmium–zinc–telluride: accuracy and Outcome (AMICO) registry demonstrated the prognostic advantage afforded by cadmium zinc telluride (CZT)-SPECT after adjustment for multiple variables like left ventricular volume and ejection fraction, coronary anatomical features, biohumoral parameters, treatment strategy and medical therapy. Of note, patients with higher Summed Stress Score (SSS >8) had a worst outcome, especially those medically treated [48].

#### 3.1.3 Previous Myocardial Infarction

Perfusion imaging identifies not only ischemia as reversible perfusion defects but also myocardial scars as fixed perfusion defects. In a study involving 3216 patients with prior myocardial infarction undergoing SPECT, 70% presented scars which were judged clinically significant in 25% of cases (>5% of myocardium with scar) [49]. Myocardial SPECT was performed in 13,555 patients with and without a history of CAD. Surprisingly, a survival benefit over medical therapy was observed with revascularization in patients with severe ischemia but without a history of CAD and with prior revascularization but not in patients with prior infarction. When patients with more extensive scar (>10% of myocardium) were excluded, increasing ischemia was associated with a survival benefit irrespective of a history of prior myocardial infarction. The findings highlighted the lesser role of ischemia compared to extensive scar and the potential harm associated with an invasive approach if no ischemia is present.

#### 3.1.4 MPI after Revascularization

Functional testing after coronary revascularization is routinely performed in clinical practice. According to observational data, 1 out of 2 to 3 patients undergoes a stress test within two years after percutaneous revascularization [50, 51, 52]. In a study involving 1848 patients undergoing revascularization mainly for acute coronary syndromes, 1 out of 8 underwent an imaging stress test even if asymptomatic. This approach led to repetition of revascularization in only <1% of patients, questioning the value of stress imaging in this setting [53]. Current data that any form of routine stress testing may improve outcome in stable CAD are limited. The European guidelines [54] give a weak recommendation for surveillance stress test at 1 year after percutaneous coronary intervention (PCI) (COR IIb, LOE C), whereas the American guidelines [55] offer no recommendations. Recently published data of the POST-PCI trial refrain from prescribing surveillance stress testing [56]. A study involving 1706 patients undergoing PCI considered at high-risk based on anatomical or clinical features were randomized to undergo stress testing at one year post intervention or to standard care. At two-year follow-up there was no difference in outcome between the two strategies [55]. Invasive diagnostic angiography and revascularization were more frequent in the group randomized to the stress test approach; however, findings showed no reduction in the primary outcome in comparison to the group that received guideline-directed medical therapy. Another study evaluated a routine stress test strategy after coronary artery bypass surgery [57] and found that ischemia was associated with worse outcome and that repeated revascularization did not modify event risk.

The risk for major adverse cardiac events (MACE) on follow-up was reported to be proportional to the magnitude of residual ischemia and that a 5% reduction in ischemia had a significant prognostic benefit [45]. The observational BASKET LATE IMAGING study [58] reported that abnormal SPECT findings 5 years after revascularization were frequent regardless of symptoms and predictive of adverse events. While re-evaluation of risk may be useful to guide intensification of medical therapy in selected high-risk patients, perfusion imaging is rarely appropriate unless symptoms or a change in clinical status occurs, especially if it is performed less than 2 years after PCI or less than 5 years after coronary artery bypass graft (CABG) surgery. Patients in which myocardial SPECT is strongly recommended have previous obstructive CAD (myocardial infarction or coronary revascularization, COR I, LOE B) or known non-obstructive CAD (COR IIa, LOE C) but only when stable chest pain persists despite optimal medical therapy [31, 54]. Guidelines recognize the role of perfusion imaging for risk assessment after percutaneous or surgical revascularization in patients with incomplete revascularization, left main artery or proximal left anterior descending disease, diabetes or other high-risk factors [59, 60]. In patients with limiting angina and insufficient response to optimized medical therapy or significant anatomical or functional CAD (>10% ischemic myocardium) revascularization is recommended (COR I, LOE A/B) [54].

### 3.2 Suspected Acute Coronary Syndrome (ACS)

Perfusion imaging has demonstrated its utility in the emergency department for triage decision making. Early resting SPECT has become common practice in the United States [61]. In the early assessment of acute chest pain, resting SPECT can be appropriately performed in patients with ongoing symptoms or symptom resolution within 3 hours after evaluation and in the absence of other findings suggestive of ACS like ECG abnormalities or elevated first troponin levels [62]. In this setting, normal resting perfusion SPECT showed a high negative predictive value for acute infarction and short-term cardiac events, so that these patients can be safety discharged [63]. Acute resting SPECT proved a cost-effective approach [64, 65]. If symptoms have resolved hours before evaluation in the emergency department (3 hours or more in clinical trials), rest SPECT may be performed but the test could result insensitive to perfusion defects. When ACS is still suspected after serial ECG and troponin result negative or borderline for ACS, and the patient does not qualify for ‘rule-out’ or ‘rule-in’, non-invasive imaging using stress testing targeting myocardial ischemia or CCTA is recommended before deciding on an invasive approach (COR I, LOE B) [66, 67]. In such cases, stress-rest SPECT or a stress-only protocol is considered appropriate and safe to rule-out ACS [18, 25, 62]. In patients with known CAD and new-onset or worsening symptoms, stress testing is indicated (COR IIa, LOE B) since rest SPECT cannot distinguish between chronic and acute ischemia. Direct invasive coronary angiography (ICA) is recommended only when there is previously documented significant left main or proximal left anterior descending or multivessel CAD or in patients with previous coronary revascularization (COR I, LOE A).

### 3.3 Before Noncardiac Surgery

**Fig. 4.**
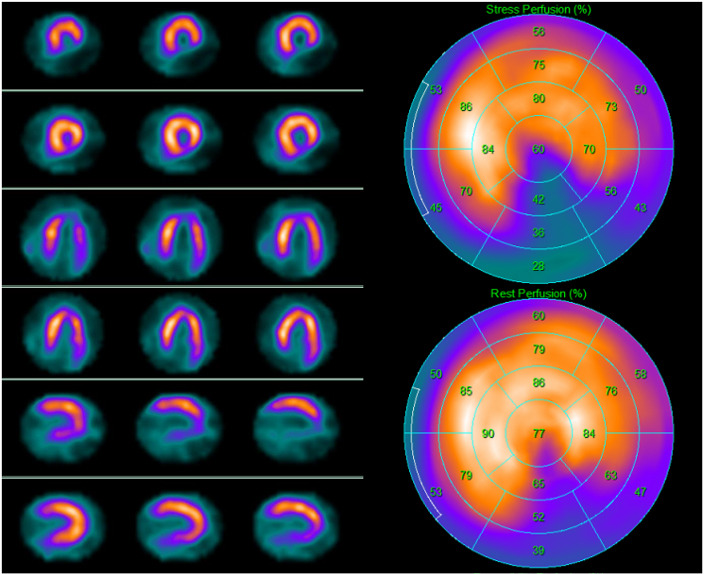
**A 69-year-old asymptomatic man with multiple cardiovascular risk factors underwent 99mTc-tetrofosmin cardiac single photon emission computed tomography (SPECT) screening for myocardial ischemia before kidney transplantation**. SPECT revealed a partially reversible inferior defect. Subsequent invasive coronary angiography revealed severe stenosis of the posterior descending artery, which was treated with angioplasty. Left side top row: stress-rest short axis; stress-rest horizontal long axis; stress-rest vertical long axis; right side: polar map of stress (upper image) and rest perfusion (lower image).

The role of stress testing in preoperative risk assessment of patients before noncardiac surgery has been extensively demonstrated [68, 69, 70]. Moderate to severe myocardial ischemia is a sensitive marker of increased risk of perioperative MACE. Normal SPECT findings portend a high negative predictive value for perioperative cardiac events, while detection of scars has a low positive predictive value. Due to the underlying CAD, however, long-term prognosis is worse in these patients (Fig. 4).

In a meta-analysis of ten studies with pharmacological stress SPECT, the 30-day MACE rates were 1% in patients with normal test results, 7% and 9% in those with fixed and reversible perfusion defects, respectively, with a higher event rate in those with at least two reversible defects [71].

According to American guidelines, recommendations for stress imaging are limited to patients with elevated surgical risk, performing exercise stress imaging in patients with excellent (>10 METs) (COR IIa, LOE B) or moderate to good (METs 4-10) (COR IIb, LOE B) functional capacity, pharmacological stress imaging in those with poor functional capacity (<4 METs) when test results are used to change management (COR IIa, LOE B) [72]. In this setting, the European guidelines include the role of clinical risk factors [73]. Stress imaging is recommended before high-risk surgery in patients with one or more clinical risk factors or a high likelihood of CAD (PTP >15% or two or more cardiovascular risk factors, resting ECG changes or left ventricular dysfunction suggestive of CAD) and poor functional capacity (COR I, LOE B). Stress imaging should also be considered in patients with poor functional capacity and previous coronary revascularization (COR IIa, LOE C) [74].

### 3.4 Heart Failure 

**Fig. 5.**
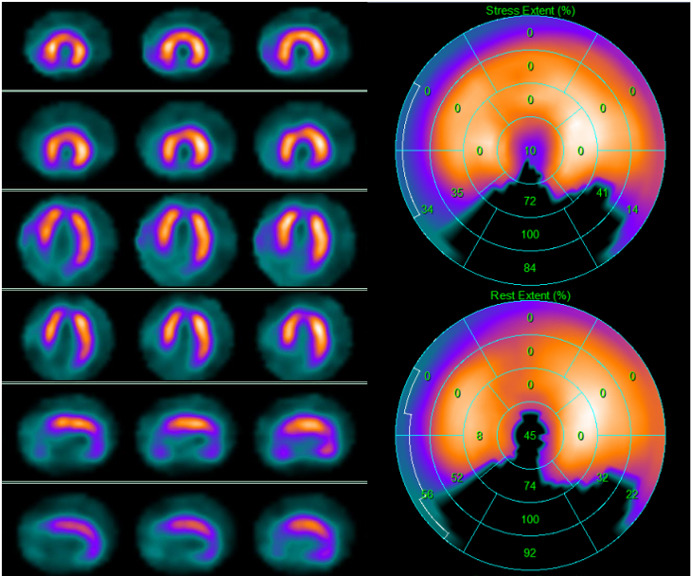
**99mTc-tetrofosmin cardiac single photon emission computed tomography (SPECT) images in a 67-year-old man with dyspnea on exertion and new-onset left ventricular dysfunction with inferior wall akinesia at resting echocardiography**. SPECT revealed on the inferior wall a scar due to a previous silent myocardial infarction. Left side top row: stress-rest short axis; stress-rest horizontal long axis; stress-rest vertical long axis; right side: polar map of stress (upper image) and rest perfusion (lower image).

CAD is a major contributor to heart failure worldwide [75]. Tests detecting CAD help clinicians understand the etiology of heart failure and guide patient management in relation to symptoms and prognosis improvement (COR IIa, LOE B) [76]. New or worsening symptoms of heart failure may be associated with myocardial ischemia (Fig. 5). Severely dysfunctional but viable myocardium (hibernating myocardium) is associated with poor outcome, but appropriate revascularization may ameliorate the prognosis [77, 78, 79]. The recently published results of the Surgical Treatment for Ischemic Heart Failure Extension Study (STICHES) showed that patients with ischemic heart disease who underwent CABG surgery had a better prognosis than those who received medical therapy alone at 10-year follow-up [80]. Stress imaging (SPECT or positron-emission-tomography [PET], stress echocardiography, cardiac magnetic resonance) may be considered in patients with CAD who are eligible for coronary revascularization in which the aim should be the detection of myocardial ischemia and viability (COR IIb, LOE B) [76, 81]. Debate continues to surround the management of ischemic left ventricular dysfunction because ischemia, hibernation, viability, scar, and remodeling are variably involved, and it is unclear how to identify the patients who may gain benefit from revascularization in terms of prognosis.

In clinical practice, the evidence for myocardial viability is necessary, given that superior survival rates afforded by revascularization strategy over medical therapy have been reported only in patients with hibernating myocardium [77, 82, 83]. In the STICH trial, however, the degree of left ventricular systolic dysfunction and remodeling and the number of stenotic coronary arteries appeared to be stronger determinants of the benefit of revascularization than myocardial viability [84]. In the prespecified viability sub-study of the STICH trial, myocardial viability was associated with a modest improvement in left ventricular systolic function, irrespective of treatment, albeit not associated with a long-term benefit over surgical revascularization at 10-year follow-up [85]. In the PPAR-2 trial, assessment of ischemia was associated with incremental benefit over viability, especially in patients with mild to moderate CAD [86]. The stress-rest SPECT study mentioned above revealed that the extent of ischemic myocardium (>10% inducible ischemia) identified patients who might benefit from revascularization but only when there was no extensive scar (<10% of total myocardium) [49]. A subsequent PET study addressed the entire issue and examined the ability of measures of inducible ischemia and hibernation in identifying optimal therapeutic strategies in patients with ischemic left ventricular dysfunction [79]. An association was found between the extent and severity of hibernating myocardium, treatment strategy, and survival, and that prognosis improves with early revascularization with at least 10% myocardium hibernating and the survival rate improves proportionally as the percent of hibernating myocardium further increases.

## 4. SPECT versus PET Myocardial Perfusion Imaging

In nuclear medicine, both SPECT and PET modalities can be used to evaluate myocardial perfusion for diagnosing CAD. A higher pooled mean sensitivity for significant CAD has been reported for PET in comparison to SPECT, but the respective value of SPECT and PET in terms of specificity is less defined. The EVINCI study showed higher sensitivity and specificity of PET compared to SPECT in the detection of coronary stenosis (81% versus 73% and 89% versus 67%, respectively) in patients with an intermediate likelihood of CAD [87]. In the PACIFIC study, patients with suspected CAD underwent CCTA, PET, and SPECT, followed by ICA with fractional flow reserve (FFR) measurements [88]. Surprisingly, SPECT resulted noninferior to PET in specificity for significant CAD. On comparison of the three modalities, CCTA showed the highest sensitivity (90%), whereas SPECT and PET showed higher specificity compared to CCTA (94% and 84% versus 60%, respectively); PET showed the highest diagnostic accuracy overall (85%), whereas CCTA and SPECT showed similar accuracy (74% and 77%, respectively). In the PACIFIC 2 study, patients with a history of myocardial infarction or percutaneous revascularization underwent SPECT, PET, and cardiac magnetic resonance, followed by ICA with FFR. PET had the highest sensitivity for hemodynamically significant CAD, whereas specificity and diagnostic accuracy did not differ between the three imaging modalities [89].

**Fig. 6.**
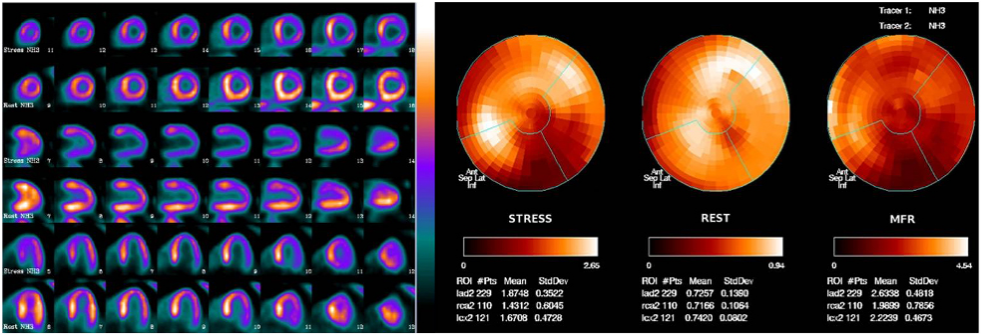
**Myocardial perfusion PET with ^13^N-ammonia of an 81-year-old male with exertional angina**. Stress images (left side) showed severe reduction in tracer uptake at inferior and antero-lateral ventricular segments and at the apex, and moderate reduction in uptake at distal infero-septal and basal anterior segments. Rest images (left side) showed mild reduced tracer uptake at the basal and middle antero-lateral segments. The measure of quantitative stress and rest myocardial blood flow (MBF) (right side) showed a mild reduced resting MBF with decreased maximal hyperaemic MBF and myocardial flow reserve (MFR). The subsequent invasive coronary angiography revealed severe stenoses of the right coronary artery and of the left circumflex coronary artery, and non-significative stenosis of the left anterior descending coronary artery. Left side (from top to bottom): stress-rest short axis; horizontal long axis; vertical long axis. Right side (from left to right): polar map of stress and rest quantitative perfusion and of myocardial flow reserve (MFR).

In addition to quantifying perfusion defects and ventricular function, PET MPI is the gold standard for the non-invasive evaluation of absolute myocardial blood flow (MBF) and myocardial blood flow reserve (MFR) (Fig. 6). MFR (the ratio between maximal hyperemic flow and resting myocardial flow) reflects the global hemodynamic effect of CAD, including coronary artery stenosis and microvascular dysfunction. PET-derived MFR estimation has been strongly associated with prognosis and can help in guiding treatment strategies in patients with CAD [90]. The ability to assess MBF and MFR is a considerable advantage of PET over SPECT. With the introduction of the new cadmium, zinc, tellurium (CZT) technology to SPECT, dynamic acquisition can be performed to assess quantitative flow indices (MBF and MFR) similar to dynamic PET study [2]. Supporting evidence is growing [91], which will probably make MFR study with SPECT technology more available due to the widespread use and lower cost of SPECT compared to PET. In this setting, assessment of absolute MBF and MFR with SPECT is promising and may also fill the gap for the proper diagnosis of multivessel disease, which has been historically considered a main limitation of SPECT [19, 92]. More data are needed before the technique becomes routine use; nonetheless, the available data are highly encouraging.

## 5. Multimodality Approach 

Diagnosis of CAD relies on the detection of atherosclerosis extension and severity and of myocardial ischemia, the anatomical and functional substrates of CAD, respectively. CT has become a primary tool for CAD detection by coronary artery calcium (CAC) quantification and direct coronary artery visualization and stenosis quantification by CCTA, whereas SPECT can quantify the hemodynamic consequences of anatomic CT findings. Since the correlation between CAC, coronary artery stenosis, and myocardial perfusion is not linear [93, 94, 95], these approaches are considered complementary rather than mutually exclusive. SPECT combined with CT has gained growing interest in the concept of hybrid imaging.

CT is routinely performed in combination a perfusion study to manage attenuation of SPECT photons in the body and improve perfusion imaging quality. This procedure is called CT-based attenuation correction and is recommended by European Association of Nuclear Medicine (EANM) guidelines [16]. It provides a map of the attenuation coefficients based on the Hounsfield unit of a low voltage CT scan. The additional patient effective dose is very low, so these CT scans can be used to approximate the extent of coronary calcification [96]. Alternatively, CT for CAC scoring can be performed [97] and then used for attenuation correction [16]. Furthermore, SPECT and CCTA datasets can be fused to create a single fused hybrid image that integrates anatomical and functional information.

### 5.1 SPECT and CAC Score 

**Fig. 7.**
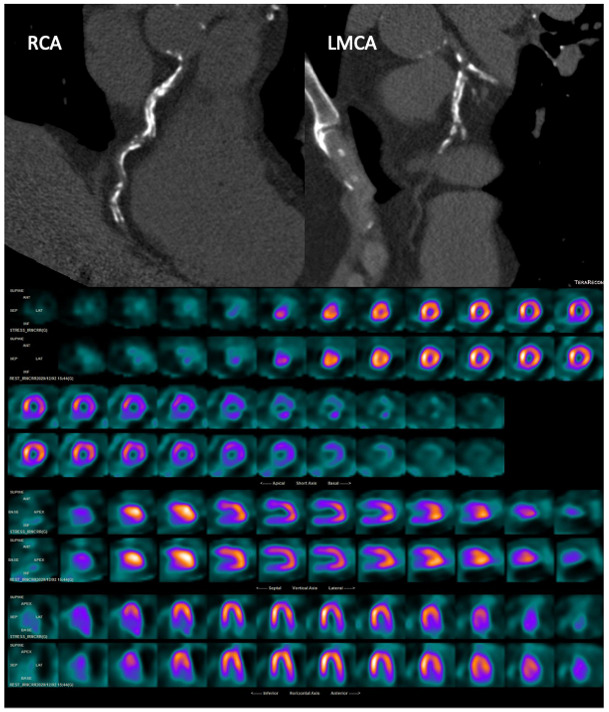
**A 77-year-old asymptomatic man with multiple cardiovascular risk factors underwent CT with CAC scoring and subsequent 99mTc-tetrofosmin cardiac SPECT**. CT images (top) showed a very high CAC score (2129 HU) in the three main vessels, whereas myocardial perfusion SPECT (bottom) showed a partially reversible defect in the basal and middle anterior segments and in the basal and middle inferior segments. RCA, right coronary artery; LMCA, left main coronary artery. Myocardial perfusion SPECT images: stress-rest short axis; stress-rest horizontal long axis; stress-rest vertical long axis.

Although myocardial ischemia is a potent predictor of cardiac events, most events occur in patients with a normal functional test. Combining a patient’s CAC score with perfusion imaging findings increases risk stratification efficacy in patients with and without myocardial ischemia [98, 99] (Fig. 7).

The CAC score is a low-radiation and low-cost non-invasive estimate of coronary atherosclerotic plaque burden, though it does not reflect obstructive CAD or ischemia. A zero CAC score demonstrated very high sensitivity to rule out obstructive CAD and to predict very low events rates in asymptomatic and symptomatic patients with suspected CAD [43, 100, 101, 102]. Differently, a positive CAC score is proportionally related to the frequency of obstructive CAD and inducible ischemia, also when calcifications are not directly associated with the degree of luminal or functional coronary artery stenosis. CAC score was found equal to CCTA to predict MPI alterations [103]. A meta-analysis [104] of studies involving asymptomatic and symptomatic patients reported a prevalence of ischemia of 6.6% for CAC-zero patients, 8.5% for CAC score 1 to 100, 10.5% for CAC score 100 to 399, and 23.6% for CAC score ≥400. The association between CAC score range and frequency of ischemia [4] suggests that stress tests can be performed in patients with symptoms suggestive of CAD and a CAC score >100 [105].

Given the strengths and the limitations of CAC evaluation and SPECT MPI (i.e., high sensitivity of CAC score for detecting CAD and high specificity of functional tests for obstructive CAD), a combined strategy dictates that a zero CAC score may rule out patients, especially those with low-intermediate PTP and atypical chest pain, whereas MPI or other functional tests may identify patients with a positive CAC score at higher risk of events [43]. CT for CAC scoring is now recommended in patients with stable symptoms categorized as low-risk (COR IIa, LOE B) [31].

In patients with suspected CAD, the complementary use of CAC score and SPECT-MPI may: improve the assessment of PTP of CAD in initial diagnostic work-up and help in selecting patients for stress testing; improve cardiac risk stratification, since risk increases in patients with abnormal MPI findings who also have CAC abnormality [106, 107] and in patients with normal SPECT findings and a CAC score >400 [108]. When the two procedures are performed contemporary, the diagnostic sensitivity of SPECT may improve by identifying normal SPECT-MPI patients with subclinical atherosclerosis and improve interpretation of equivocal SPECT studies [13, 109].

The CAC score is not a diagnostic tool for obstructive CAD but rather a cardiovascular risk modifier. In diabetic patients, MPI abnormalities are more strongly associated with CAC score than with traditional risk factors [110]. For example, symptomatic patients with non-calcified obstructive plaque may have a zero CAC score but spotty or microcalcifications associated with high-risk plaques. Several studies reported that the relationship between CAC score and the likelihood of CAD is influenced by the overall clinical risk of the population, the patient’s clinical presentation and PTP [111, 112, 113, 114, 115, 116]. In patients at high risk for CAD, abnormal MPI findings were more frequent than in patients at low or intermediate risk also in those with a low CAC score [117, 118].

Finally, cardiac event risk was significantly higher in asymptomatic patients with normal SPECT-MPI when the CAC score was elevated (>400) [98]. CAC score has been proposed as a gatekeeper to identify asymptomatic subjects at high risk in which further screening with functional testing may be warranted [37] and indicated in those with a CAC score >400 or between 100 and 400 in high-risk asymptomatic patients [105]. Finally, CAC scoring has proven a robust and specific method to measure subclinical atherosclerosis and guide initiation or intensification of treatment.

### 5.2 Fusion-Hybrid SPECT/CT Imaging

**Fig. 8.**
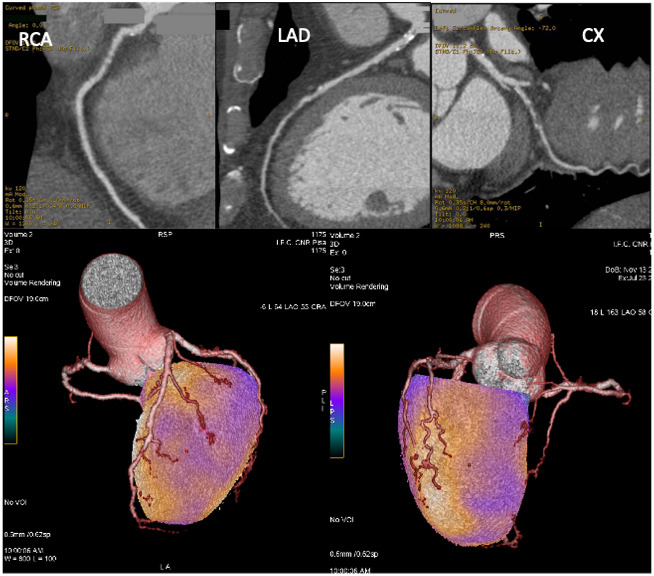
**Coronary CT angiography and hybrid myocardial perfusion SPECT/coronary CT angiography imaging of the patient described in Fig. 6**. Upper images: CCTA showed non-calcific subcritical plaques in the right coronary artery (RCA; left side) and left anterior descendent coronary artery (LAD) and a stenotic non-calcific plaque in the left circumflex artery (CX; right side). Lower images: fusion of CCTA and SPECT datasets allows the integration of functional and anatomical data, clearly indicating the relationship between the atherosclerotic plaques and ischemic areas.

The synergistic combination of myocardial perfusion SPECT imaging with CCTA offers contrast-mediated visualization of the coronary artery lumen and detects anatomical abnormalities and their functional consequences in a single setting (Fig. 8). CCTA has an excellent negative predictive value (NPV) to exclude CAD, whereas less robust is the positive predictive value (PPV) for the identification of hemodynamically significant lesions [119, 120], since CCTA tends to overestimate stenosis severity due to coronary calcification or artifacts. CCTA can also document multivessel disease, which is viewed as a weak point of SPECT imaging due to possible balanced ischemia.

Hybrid cardiac SPECT/CCTA provides superior diagnostic data compared to either stand-alone or side-by-side interpretation of SPECT and CT data sets by virtue of the spatial co-localization of a myocardial perfusion defect with a subtending coronary artery. There is substantial inter-individual variability in coronary artery anatomy and actual segmental assignment to coronary artery territories differs from that expected in more than half of patients [121]. This hybrid imaging creates a panoramic three-dimensional view by integrating volume-rendered CT data with the perfusion information from SPECT to identify the culprit lesion with higher sensitivity and specificity and optimize the final ruling of intermediate lesions and equivocal perfusion defects [122]. Since a sizeable proportion of obstructive stenosis doesn’t induce ischemia [122] and coronary stenosis of 50% to 70% may be associated with coronary flow reduction [123], the complementary information provided by the two techniques can help to exclude patients with normal coronary arteries, diagnose subclinical atherosclerosis, define the ischemic burden of specific coronary lesions, detect multivessel disease, and identify the culprit lesion [124]. In a population with a high pre-test likelihood of CAD, hybrid imaging analysis improved the PPV to 96% (versus 85% and 77% of SPECT and CCTA, respectively) and the NPV to 95% (versus 89% and 96% of SPECT and CCTA, respectively) versus a reference standard of fractional flow reserve (FFR) measurement [125].

Hybrid imaging techniques may be useful also in follow up after CABG surgery, in which coronary physiology is completely altered. When ischemia is present, identification of a single coronary artery or graft as the culprit lesion poses a great challenge. A high prevalence of perfusion defects in territories supplied by patent grafts has been reported [126]. Integrating anatomical and perfusion data through hybrid imaging is a promising tool to visualize post-operative anatomy, localize vessel stenosis and occlusions, and gain additional insights into the mechanism of ischemia [127]. Also, it has been suggested that complementary approaches may improve event prediction in patients after CABG surgery [128].

The disadvantages of additional radiation exposure and cost and time investment related to fusion SPECT/CCTA limit its routine application. Sequential use of SPECT and CCTA in selected patients may be an attractive alternative [129, 130, 131, 132]. The wider availability of image fusion software may allow the use of CCTA from external sources to create fusion images [133].

### 5.3 CT Evaluation of Myocardial Ischemia 

CT myocardial perfusion (CTP) imaging has gained interest as a diagnostic tool alternative to perfusion SPECT. The CTP protocol includes rest and stress scanning phases, as in other functional imaging techniques. CTP demonstrated good diagnostic performance in detecting ischemia defined by FFR [134] or invasive coronary angiography [135], also in patients with previous percutaneous coronary angioplasty or with severe coronary calcification [136]. The results so far are encouraging. If corroborated in studies with larger patient samples, CTP may compensate the weaknesses of CCTA. CCTA and stress CTP may be integrated to assess both anatomical and functional features; however, radiation exposure is very high and iodinated contrast agents are injected.

Another emerging CT-based technique for evaluating ischemia is CT-derived FFR. A greater prognostic benefit over angiography has been demonstrated when revascularization is guided by invasively assessed FFR [137, 138], so that a non-invasive method to measure FFR seems attractive. Data extracted from resting CCTA can be used to calculate CT-derived FFR and to directly determine the significance of a CCTA lesion. Since no additional radiation or contrast agent dose is required, it’s becoming the gold standard for the identification of flow-limiting coronary artery stenosis. The drawback is that CCTA image quality need to be very high, limiting the diagnostic accuracy in severe calcifications or previous percutaneous or surgical revascularization [139]. Further evidence from randomized clinical trials is needed before extensive use of CT-derived FFR can be recommended for clinical practice. Finally, cost-effectiveness analyses and the availability of this advanced CT technology need to be taken into account.

## 6. Conclusions

SPECT-MPI has been a preferred method for evaluating patients with known or suspected CAD. With recent advances in the management of cardiovascular risk factors and acute coronary syndromes, CAD has become a chronic condition. Because many patients referred to SPECT have a low PTP, the diagnostic accuracy of MPI for obstructive CAD tends to decline. A considerable proportion of patients undergoing ICA after stress imaging has normal coronary arteries or non-obstructive coronary stenosis [140], while most cardiac events occur in patients in which inducible ischemia has not been detected. A guideline-directed selection of patients to refer to SPECT-MPI is essential to increase test diagnostic accuracy and the benefit of testing.

It is currently assumed that the relationship between atherosclerosis, degree of coronary artery stenosis, and ischemia is dynamic across levels of lesions severity and influenced by plaque stability and microvascular and endothelial dysfunction. Consequently, alternative approaches that combine anatomical and functional studies in sequence or during the same session have been developed, including CT for CAC scoring +/– SPECT MPI in the initial evaluation of patients with low-to-intermediate PTP of CAD, or fusion SPECT/CCTA imaging to define the ischemic burden of coronary lesions, detect multivessel disease or follow-up patients after CABG surgery. The synergistic performance of CAC or CCTA and SPECT MPI has demonstrated excellent diagnostic accuracy and outcome prediction of CAD. Finally, CZT technology applied to SPECT allows assessment of quantitative flow indices similar to those provided by PET studies, further supporting the value of SPECT MPI in the multimodality imaging era.

## References

[1] Rozanski A, Miller RJH, Gransar H, Han D, Slomka P, Dey D (2022). Benefit of Early Revascularization Based on Inducible Ischemia and Left Ventricular Ejection Fraction. *Journal of the American College of Cardiology*.

[2] Liga R, Neglia D, Kusch A, Favilli B, Giorgetti A, Gimelli A (2022). Prognostic Role of Dynamic CZT Imaging in CAD Patients: Interaction Between Absolute Flow and CAD Burden. *JACC: Cardiovascular Imaging*.

[3] Miller RJH, Hu L, Gransar H, Betancur J, Eisenberg E, Otaki Y (2020). Transient ischaemic dilation and post-stress wall motion abnormality increase risk in patients with less than moderate ischaemia: analysis of the REFINE SPECT registry. *European Heart Journal - Cardiovascular Imaging*.

[4] Muscogiuri G, Guglielmo M, Serra A, Gatti M, Volpato V, Schoepf UJ (2022). Multimodality Imaging in Ischemic Chronic Cardiomyopathy. *Journal of Imaging*.

[5] Fihn SD, Gardin JM, Abrams J, Berra K, Blankenship JC, Dallas AP (2012). 2012 ACCF/AHA/ACP/AATS/PCNA/SCAI/STS Guideline for the diagnosis and management of patients with stable ischemic heart disease: a report of the American College of Cardiology Foundation/American Heart Association Task Force on Practice Guidelines, and the American College of Physicians, American Association for Thoracic Surgery, Preventive Cardiovascular Nurses Association, Society for Cardiovascular Angiography and Interventions, and Society of Thoracic Surgeons. *Journal of the American College of Cardiology*.

[6] Douglas PS, Shaw LJ (2017). SPECT, PET, and CTA-Acronyms or Better Imaging. *JAMA Cardiology*.

[7] Tonino PAL, Fearon WF, De Bruyne B, Oldroyd KG, Leesar MA, Ver Lee PN (2010). Angiographic versus functional severity of coronary artery stenoses in the FAME study fractional flow reserve versus angiography in multivessel evaluation. *Journal of the American College of Cardiology*.

[8] Al-Lamee R, Thompson D, Dehbi H, Sen S, Tang K, Davies J (2018). Percutaneous coronary intervention in stable angina (ORBITA): a double-blind, randomised controlled trial. *Lancet*.

[9] Maron DJ, Hochman JS, Reynolds HR, Bangalore S, O’Brien SM, Boden WE (2020). Initial Invasive or Conservative Strategy for Stable Coronary Disease. *The New England Journal of Medicine*.

[10] Camici PG, Crea F (2007). Coronary microvascular dysfunction. *The New England Journal of Medicine*.

[11] Naya M, Murthy VL, Foster CR, Gaber M, Klein J, Hainer J (2013). Prognostic interplay of coronary artery calcification and underlying vascular dysfunction in patients with suspected coronary artery disease. *Journal of the American College of Cardiology*.

[12] Gould KL, Lipscomb K, Hamilton GW (1974). Physiologic basis for assessing critical coronary stenosis. Instantaneous flow response and regional distribution during coronary hyperemia as measures of coronary flow reserve. *The American Journal of Cardiology*.

[13] Sinusas AJ, Shi Q, Saltzberg MT, Vitols P, Jain D, Wackers FJ (1994). Technetium-99m-tetrofosmin to assess myocardial blood flow: experimental validation in an intact canine model of ischemia. *Journal of Nuclear Medicine*.

[14] Glover DK, Ruiz M, Edwards NC, Cunningham M, Simanis JP, Smith WH (1995). Comparison between 201Tl and 99mTc sestamibi uptake during adenosine-induced vasodilation as a function of coronary stenosis severity. *Circulation*.

[15] Parker MW, Iskandar A, Limone B, Perugini A, Kim H, Jones C (2012). Diagnostic accuracy of cardiac positron emission tomography versus single photon emission computed tomography for coronary artery disease: a bivariate meta-analysis. *Circulation: Cardiovascular Imaging*.

[16] Verberne HJ, Acampa W, Anagnostopoulos C, Ballinger J, Bengel F, De Bondt P (2015). EANM procedural guidelines for radionuclide myocardial perfusion imaging with SPECT and SPECT/CT: 2015 revision. *European Journal of Nuclear Medicine and Molecular Imaging*.

[17] Kirkeeide RL, Gould KL, Parsel L (1986). Assessment of coronary stenoses by myocardial perfusion imaging during pharmacologic coronary vasodilation. VII. Validation of coronary flow reserve as a single integrated functional measure of stenosis severity reflecting all its geometric dimensions. *Journal of the American College of Cardiology*.

[18] Germano G, Kiat H, Kavanagh PB, Moriel M, Mazzanti M, Su HT (1995). Automatic quantification of ejection fraction from gated myocardial perfusion SPECT. *Journal of Nuclear Medicine*.

[19] Giubbini R, Bertoli M, Durmo R, Bonacina M, Peli A, Faggiano I (2021). Comparison between N^13^NH_3_-PET and ^99m^Tc-Tetrofosmin-CZT SPECT in the evaluation of absolute myocardial blood flow and flow reserve. *Journal of Nuclear Cardiology*.

[20] Johnson LL, Verdesca SA, Aude WY, Xavier RC, Nott LT, Campanella MW (1997). Postischemic stunning can affect left ventricular ejection fraction and regional wall motion on post-stress gated sestamibi tomograms. *Journal of the American College of Cardiology*.

[21] Sharir T, Germano G, Kavanagh PB, Lai S, Cohen I, Lewin HC (1999). Incremental prognostic value of post-stress left ventricular ejection fraction and volume by gated myocardial perfusion single photon emission computed tomography. *Circulation*.

[22] Petix NR, Sestini S, Marcucci G, Coppola A, Arena A, Nassi F (2005). Can the reversible regional wall motion abnormalities on stress gated Tc-99m sestamibi SPECT predict a future cardiac event. *Journal of Nuclear Cardiology*.

[23] Dilsizian V, Bonow RO (1992). Differential uptake and apparent 201Tl washout after thallium reinjection. Options regarding early redistribution imaging before reinjection or late redistribution imaging after reinjection. *Circulation*.

[24] Gropler RJ, Geltman EM, Sampathkumaran K, Pérez JE, Schechtman KB, Conversano A (1993). Comparison of carbon-11-acetate with fluorine-18-fluorodeoxyglucose for delineating viable myocardium by positron emission tomography. *Journal of the American College of Cardiology*.

[25] Giorgetti A, Pingitore A, Favilli B, Kusch A, Lombardi M, Marzullo P (2005). Baseline/postnitrate tetrofosmin SPECT for myocardial viability assessment in patients with postischemic severe left ventricular dysfunction: new evidence from MRI. *Journal of Nuclear Medicine*.

[26] Sciagrà R, Bisi G, Santoro GM, Rossi V, Fazzini PF (1995). Nitrate versus rest myocardial scintigraphy with technetium 99m-sestamibi: relationship of tracer uptake to regional left ventricular function and its significance in the detection of viable hibernating myocardium. *American Journal of Cardiac Imaging*.

[27] Virani SS, Alonso A, Benjamin EJ, Bittencourt MS, Callaway CW, Carson AP (2020). Heart Disease and Stroke Statistics-2020 Update: A Report From the American Heart Association. *Circulation*.

[28] Lloyd-Jones D, Adams RJ, Brown TM, Carnethon M, Dai S, WRITING GROUP MEMBERS (2010). Heart disease and stroke statistics–2010 update: a report from the American Heart Association. *Circulation*.

[29] Marcassa C, Bax JJ, Bengel F, Hesse B, Petersen CL, Reyes E (2008). Clinical value, cost-effectiveness, and safety of myocardial perfusion scintigraphy: a position statement. *European Heart Journal*.

[30] Knuuti J, Wijns W, Saraste A, Capodanno D, Barbato E, Funck-Brentano C (2020). 2019 ESC Guidelines for the diagnosis and management of chronic coronary syndromes. *European Heart Journal*.

[31] Gulati M, Levy PD, Mukherjee D, Amsterdam E, Bhatt DL, Writing Committee Members (2021). 2021 AHA/ACC/ASE/CHEST/SAEM/SCCT/SCMR Guideline for the Evaluation and Diagnosis of Chest Pain: A Report of the American College of Cardiology/American Heart Association Joint Committee on Clinical Practice Guidelines. *Journal of the American College of Cardiology*.

[32] Fihn SD, Blankenship JC, Alexander KP, Bittl JA, Byrne JG, Fletcher BJ (2014). 2014 ACC/AHA/AATS/PCNA/SCAI/STS focused update of the guideline for the diagnosis and management of patients with stable ischemic heart disease: a report of the American College of Cardiology/American Heart Association Task Force on Practice Guidelines, and the American Association for Thoracic Surgery, Preventive Cardiovascular Nurses Association, Society for Cardiovascular Angiography and Interventions, and Society of Thoracic Surgeons. *Journal of the American College of Cardiology*.

[33] Juarez-Orozco LE, Saraste A, Capodanno D, Prescott E, Ballo H, Bax JJ (2019). Impact of a decreasing pre-test probability on the performance of diagnostic tests for coronary artery disease. *European Heart Journal - Cardiovascular Imaging*.

[34] Underwood SR, Anagnostopoulos C, Cerqueira M, Ell PJ, Flint EJ, Harbinson M (2004). Myocardial perfusion scintigraphy: the evidence. *European Journal of Nuclear Medicine and Molecular Imaging*.

[35] Karthikeyan G, Guzic Salobir B, Jug B, Devasenapathy N, Alexanderson E, Vitola J (2017). Functional compared to anatomical imaging in the initial evaluation of patients with suspected coronary artery disease: An international, multi-center, randomized controlled trial (IAEA-SPECT/CTA study). *Journal of Nuclear Cardiology*.

[36] Shaw LJ, Iskandrian AE (2004). Prognostic value of gated myocardial perfusion SPECT. *Journal of Nuclear Cardiology*.

[37] Acampa W, Gaemperli O, Gimelli A, Knaapen P, Schindler TH, Verberne HJ (2015). Role of risk stratification by SPECT, PET, and hybrid imaging in guiding management of stable patients with ischaemic heart disease: expert panel of the EANM cardiovascular committee and EACVI. *European Heart Journal. Cardiovascular Imaging*.

[38] Reeh J, Therming CB, Heitmann M, Højberg S, Sørum C, Bech J (2019). Prediction of obstructive coronary artery disease and prognosis in patients with suspected stable angina. *European Heart Journal*.

[39] Foldyna B, Udelson JE, Karády J, Banerji D, Lu MT, Mayrhofer T (2019). Pretest probability for patients with suspected obstructive coronary artery disease: re-evaluating Diamond-Forrester for the contemporary era and clinical implications: insights from the PROMISE trial. *European Heart Journal - Cardiovascular Imaging*.

[40] Genders TSS, Steyerberg EW, Hunink MGM, Nieman K, Galema TW, Mollet NR (2012). Prediction model to estimate presence of coronary artery disease: retrospective pooled analysis of existing cohorts. *British Medical Journal*.

[41] Baskaran L, Danad I, Gransar H, Ó Hartaigh B, Schulman-Marcus J, Lin FY (2019). A Comparison of the Updated Diamond-Forrester, CAD Consortium, and CONFIRM History-Based Risk Scores for Predicting Obstructive Coronary Artery Disease in Patients With Stable Chest Pain: The SCOT-HEART Coronary CTA Cohort. *JACC: Cardiovascular Imaging*.

[42] Genders TSS, Coles A, Hoffmann U, Patel MR, Mark DB, Lee KL (2018). The External Validity of Prediction Models for the Diagnosis of Obstructive Coronary Artery Disease in Patients With Stable Chest Pain: Insights From the PROMISE Trial. *JACC: Cardiovascular Imaging*.

[43] Budoff MJ, Mayrhofer T, Ferencik M, Bittner D, Lee KL, Lu MT (2017). Prognostic Value of Coronary Artery Calcium in the PROMISE Study (Prospective Multicenter Imaging Study for Evaluation of Chest Pain). *Circulation*.

[44] Fordyce CB, Douglas PS, Roberts RS, Hoffmann U, Al-Khalidi HR, Patel MR (2017). Identification of Patients With Stable Chest Pain Deriving Minimal Value From Noninvasive Testing: The PROMISE Minimal-Risk Tool, A Secondary Analysis of a Randomized Clinical Trial. *JAMA Cardiology*.

[45] Hachamovitch R, Hayes SW, Friedman JD, Cohen I, Berman DS (2003). Comparison of the short-term survival benefit associated with revascularization compared with medical therapy in patients with no prior coronary artery disease undergoing stress myocardial perfusion single photon emission computed tomography. *Circulation*.

[46] Shaw LJ, Berman DS, Maron DJ, Mancini GBJ, Hayes SW, Hartigan PM (2008). Optimal medical therapy with or without percutaneous coronary intervention to reduce ischemic burden: results from the Clinical Outcomes Utilizing Revascularization and Aggressive Drug Evaluation (COURAGE) trial nuclear substudy. *Circulation*.

[47] Tarakji KG, Brunken R, McCarthy PM, Al-Chekakie MO, Abdel-Latif A, Pothier CE (2006). Myocardial viability testing and the effect of early intervention in patients with advanced left ventricular systolic dysfunction. *Circulation*.

[48] Gimelli A, Pugliese NR, Buechel RR, Coceani M, Clemente A, Kaufmann PA (2022). Myocardial perfusion scintigraphy for risk stratification of patients with coronary artery disease: the AMICO registry. *European Heart Journal - Cardiovascular Imaging*.

[49] Hachamovitch R, Rozanski A, Shaw LJ, Stone GW, Thomson LEJ, Friedman JD (2011). Impact of ischaemia and scar on the therapeutic benefit derived from myocardial revascularization vs. medical therapy among patients undergoing stress-rest myocardial perfusion scintigraphy. *European Heart Journal*.

[50] Kini V, Parks M, Liu W, Waldo SW, Ho PM, Bradley SM (2022). Patient Symptoms and Stress Testing After Elective Percutaneous Coronary Intervention in the Veterans Affairs Health Care System. *JAMA Network Open*.

[51] Bagai A, Eberg M, Koh M, Cheema AN, Yan AT, Dhoot A (2017). Population-Based Study on Patterns of Cardiac Stress Testing After Percutaneous Coronary Intervention. *Circulation: Cardiovascular Quality and Outcomes*.

[52] Dhoot A, Liu S, Savu A, Cheema ZM, Welsh RC, Bainey KR (2020). Cardiac Stress Testing After Coronary Revascularization. *The American Journal of Cardiology*.

[53] Peterson T, Askew JW, Bell M, Crusan D, Hodge D, Gibbons RJ (2014). Low yield of stress imaging in a population-based study of asymptomatic patients after percutaneous coronary intervention. *Circulation: Cardiovascular Imaging*.

[54] Neumann F, Sousa-Uva M, Ahlsson A, Alfonso F, Banning AP, Benedetto U (2019). 2018 ESC/EACTS Guidelines on myocardial revascularization. *European Heart Journal*.

[55] Lawton JS, Tamis-Holland JE, Bangalore S, Bates ER, Beckie TM, Writing Committee Members (2022). 2021 ACC/AHA/SCAI Guideline for Coronary Artery Revascularization: A Report of the American College of Cardiology/American Heart Association Joint Committee on Clinical Practice Guidelines. *Journal of the American College of Cardiology*.

[56] Park D, Kang D, Ahn J, Yun S, Yoon Y, Hur S (2022). Routine Functional Testing or Standard Care in High-Risk Patients after PCI. *The New England Journal of Medicine*.

[57] Harb SC, Cook T, Jaber WA, Marwick TH (2012). Exercise testing in asymptomatic patients after revascularization: are outcomes altered. *Archives of Internal Medicine*.

[58] Zellweger MJ, Fahrni G, Ritter M, Jeger RV, Wild D, Buser P (2014). Prognostic value of ”routine” cardiac stress imaging 5 years after percutaneous coronary intervention: the prospective long-term observational BASKET (Basel Stent Kosteneffektivitäts Trial) LATE IMAGING study. *JACC: Cardiovascular Interventions*.

[59] Eagle KA, Guyton RA, Davidoff R, Edwards FH, Ewy GA, Gardner TJ (2004). ACC/AHA 2004 guideline update for coronary artery bypass graft surgery: summary article. A report of the American College of Cardiology/American Heart Association Task Force on Practice Guidelines (Committee to Update the 1999 Guidelines for Coronary Artery Bypass Graft Surgery). *Journal of the American College of Cardiology*.

[60] Smith SC, Dove JT, Jacobs AK, Kennedy JW, Kereiakes D, Kern MJ (2001). ACC/AHA guidelines of percutaneous coronary interventions (revision of the 1993 PTCA guidelines)–executive summary. A report of the American College of Cardiology/American Heart Association Task Force on Practice Guidelines (committee to revise the 1993 guidelines for percutaneous transluminal coronary angioplasty). *Journal of the American College of Cardiology*.

[61] Heller GV, Stowers SA, Hendel RC, Herman SD, Daher E, Ahlberg AW (1998). Clinical value of acute rest technetium-99m tetrofosmin tomographic myocardial perfusion imaging in patients with acute chest pain and nondiagnostic electrocardiograms. *Journal of the American College of Cardiology*.

[62] Rybicki FJ, Udelson JE, Peacock WF, Goldhaber SZ, Isselbacher EM, Kazerooni E (2016). 2015 ACR/ACC/AHA/AATS/ACEP/ASNC/NASCI/SAEM/SCCT/ SCMR/SCPC/SNMMI/STR/STS Appropriate Utilization of Cardiovascular Imaging in Emergency Department Patients With Chest Pain: A Joint Document of the American College of Radiology Appropriateness Criteria Committee and the American College of Cardiology Appropriate Use Criteria Task Force. *Journal of the American College of Cardiology*.

[63] Udelson JE, Beshansky JR, Ballin DS, Feldman JA, Griffith JL, Handler J (2002). Myocardial perfusion imaging for evaluation and triage of patients with suspected acute cardiac ischemia: a randomized controlled trial. *The Journal of the American Medical Association*.

[64] Forberg JL, Hilmersson CE, Carlsson M, Arheden H, Björk J, Hjalte K (2009). Negative predictive value and potential cost savings of acute nuclear myocardial perfusion imaging in low risk patients with suspected acute coronary syndrome: a prospective single blinded study. *BMC Emergency Medicine*.

[65] Taban Sadeghi M, Mahmoudian B, Ghaffari S, Moharamzadeh P, Ala A, Pourafkari L (2019). Value of early rest myocardial perfusion imaging with SPECT in patients with chest pain and non-diagnostic ECG in emergency department. *The International Journal of Cardiovascular Imaging*.

[66] Collet J, Thiele H, Barbato E, Barthélémy O, Bauersachs J, Bhatt DL (2021). 2020 ESC Guidelines for the management of acute coronary syndromes in patients presenting without persistent ST-segment elevation. *European Heart Journal*.

[67] Amsterdam EA, Wenger NK, Brindis RG, Casey DE, Ganiats TG, Holmes DR (2014). 2014 AHA/ACC Guideline for the Management of Patients with Non-ST-Elevation Acute Coronary Syndromes: a report of the American College of Cardiology/American Heart Association Task Force on Practice Guidelines. *Journal of the American College of Cardiology*.

[68] Harafuji K, Chikamori T, Kawaguchi S, Obitsu Y, Ito S, Igarashi Y (2005). Value of pharmacologic stress myocardial perfusion imaging for preoperative risk stratification for aortic surgery. *Circulation Journal*.

[69] Cohen MC, Siewers AE, Dickens JD, Hill T, Muller JE (2003). Perioperative and long-term prognostic value of dipyridamole Tc-99m sestamibi myocardial tomography in patients evaluated for elective vascular surgery. *Journal of Nuclear Cardiology*.

[70] Stratmann HG, Younis LT, Wittry MD, Amato M, Miller DD (1996). Dipyridamole technetium-99m sestamibi myocardial tomography in patients evaluated for elective vascular surgery: prognostic value for perioperative and late cardiac events. *American Heart Journal*.

[71] Shaw LJ, Eagle KA, Gersh BJ, Miller DD (1996). Meta-analysis of intravenous dipyridamole-thallium-201 imaging (1985 to 1994) and dobutamine echocardiography (1991 to 1994) for risk stratification before vascular surgery. *Journal of the American College of Cardiology*.

[72] Fleisher LA, Fleischmann KE, Auerbach AD, Barnason SA, Beckman JA, Bozkurt B (2014). 2014 ACC/AHA guideline on perioperative cardiovascular evaluation and management of patients undergoing noncardiac surgery: a report of the American College of Cardiology/American Heart Association Task Force on practice guidelines. *Journal of the American College of Cardiology*.

[73] Ford MK, Beattie WS, Wijeysundera DN (2010). Systematic review: prediction of perioperative cardiac complications and mortality by the revised cardiac risk index. *Annals of Internal Medicine*.

[74] Halvorsen S, Mehilli J, Cassese S, Hall TS, Abdelhamid M, Barbato E (2022). 2022 ESC Guidelines on cardiovascular assessment and management of patients undergoing non-cardiac surgery. *European Heart Journal*.

[75] Groenewegen A, Rutten FH, Mosterd A, Hoes AW (2020). Epidemiology of heart failure. *European Journal of Heart Failure*.

[76] Heidenreich PA, Bozkurt B, Aguilar D, Allen LA, Byun JJ, Colvin MM (2022). 2022 AHA/ACC/HFSA Guideline for the Management of Heart Failure: A Report of the American College of Cardiology/American Heart Association Joint Committee on Clinical Practice Guidelines. *Journal of the American College of Cardiology*.

[77] Allman KC, Shaw LJ, Hachamovitch R, Udelson JE (2002). Myocardial viability testing and impact of revascularization on prognosis in patients with coronary artery disease and left ventricular dysfunction: a meta-analysis. *Journal of the American College of Cardiology*.

[78] Orlandini A, Castellana N, Pascual A, Botto F, Cecilia Bahit M, Chacon C (2015). Myocardial viability for decision-making concerning revascularization in patients with left ventricular dysfunction and coronary artery disease: a meta-analysis of non-randomized and randomized studies. *International Journal of Cardiology*.

[79] Ling LF, Marwick TH, Flores DR, Jaber WA, Brunken RC, Cerqueira MD (2013). Identification of therapeutic benefit from revascularization in patients with left ventricular systolic dysfunction: inducible ischemia versus hibernating myocardium. *Circulation: Cardiovascular Imaging*.

[80] Velazquez EJ, Lee KL, Jones RH, Al-Khalidi HR, Hill JA, Panza JA (2016). Coronary-Artery Bypass Surgery in Patients with Ischemic Cardiomyopathy. *The New England Journal of Medicine*.

[81] McDonagh TA, Metra M, Adamo M, Gardner RS, Baumbach A, Böhm M (2021). 2021 ESC Guidelines for the diagnosis and treatment of acute and chronic heart failure. *European Heart Journal*.

[82] Camici PG, Prasad SK, Rimoldi OE (2008). Stunning, hibernation, and assessment of myocardial viability. *Circulation*.

[83] Inaba Y, Chen JA, Bergmann SR (2010). Quantity of viable myocardium required to improve survival with revascularization in patients with ischemic cardiomyopathy: A meta-analysis. *Journal of Nuclear Cardiology*.

[84] Panza JA, Velazquez EJ, She L, Smith PK, Nicolau JC, Favaloro RR (2014). Extent of coronary and myocardial disease and benefit from surgical revascularization in ischemic LV dysfunction [Corrected]. *Journal of the American College of Cardiology*.

[85] Panza JA, Ellis AM, Al-Khalidi HR, Holly TA, Berman DS, Oh JK (2019). Myocardial Viability and Long-Term Outcomes in Ischemic Cardiomyopathy. *The New England Journal of Medicine*.

[86] Beanlands RSB, Nichol G, Huszti E, Humen D, Racine N, Freeman M (2007). F-18-fluorodeoxyglucose positron emission tomography imaging-assisted management of patients with severe left ventricular dysfunction and suspected coronary disease: a randomized, controlled trial (PARR-2). *Journal of the American College of Cardiology*.

[87] Neglia D, Rovai D, Caselli C, Pietila M, Teresinska A, Aguadé-Bruix S (2015). Detection of significant coronary artery disease by noninvasive anatomical and functional imaging. *Circulation: Cardiovascular Imaging*.

[88] Danad I, Raijmakers PG, Driessen RS, Leipsic J, Raju R, Naoum C (2017). Comparison of Coronary CT Angiography, SPECT, PET, and Hybrid Imaging for Diagnosis of Ischemic Heart Disease Determined by Fractional Flow Reserve. *JAMA Cardiology*.

[89] Driessen RS, van Diemen PA, Raijmakers PG, Knuuti J, Maaniitty T, Underwood SR (2022). Functional stress imaging to predict abnormal coronary fractional flow reserve: the PACIFIC 2 study. *European Heart Journal*.

[90] Patel KK, Spertus JA, Chan PS, Sperry BW, Al Badarin F, Kennedy KF (2020). Myocardial blood flow reserve assessed by positron emission tomography myocardial perfusion imaging identifies patients with a survival benefit from early revascularization. *European Heart Journal*.

[91] Okada RD, Glover D, Gaffney T, Williams S (1988). Myocardial kinetics of technetium-99m-hexakis-2-methoxy-2-methylpropyl-isonitrile. *Circulation*.

[92] Błaszczyk M, Adamczewski Z, Płachcińska A (2021). Capabilities of Modern Semiconductor Gamma Cameras in Radionuclide Diagnosis of Coronary Artery Disease. *Diagnostics*.

[93] Rosman J, Shapiro M, Pandey A, VanTosh A, Bergmann SR (2006). Lack of correlation between coronary artery calcium and myocardial perfusion imaging. *Journal of Nuclear Cardiology*.

[94] Schuijf JD, Wijns W, Jukema JW, Atsma DE, de Roos A, Lamb HJ (2006). Relationship between noninvasive coronary angiography with multi-slice computed tomography and myocardial perfusion imaging. *Journal of the American College of Cardiology*.

[95] van Werkhoven JM, Schuijf JD, Gaemperli O, Jukema JW, Boersma E, Wijns W (2009). Prognostic value of multislice computed tomography and gated single-photon emission computed tomography in patients with suspected coronary artery disease. *Journal of the American College of Cardiology*.

[96] Einstein AJ, Johnson LL, Bokhari S, Son J, Thompson RC, Bateman TM (2010). Agreement of visual estimation of coronary artery calcium from low-dose CT attenuation correction scans in hybrid PET/CT and SPECT/CT with standard Agatston score. *Journal of the American College of Cardiology*.

[97] Camoni L, Santos A, Attard M, Mada MO, Pietrzak AK, Rac S (2020). Best practice for the nuclear medicine technologist in CT-based attenuation correction and calcium score for nuclear cardiology. *European Journal of Hybrid Imaging*.

[98] Chang SM, Nabi F, Xu J, Peterson LE, Achari A, Pratt CM (2009). The coronary artery calcium score and stress myocardial perfusion imaging provide independent and complementary prediction of cardiac risk. *Journal of the American College of Cardiology*.

[99] Engbers EM, Timmer JR, Ottervanger JP, Mouden M, Knollema S, Jager PL (2016). Prognostic Value of Coronary Artery Calcium Scoring in Addition to Single-Photon Emission Computed Tomographic Myocardial Perfusion Imaging in Symptomatic Patients. *Circulation: Cardiovascular Imaging*.

[100] Sarwar A, Shaw LJ, Shapiro MD, Blankstein R, Hoffmann U, Cury RC (2009). Diagnostic and prognostic value of absence of coronary artery calcification. *JACC: Cardiovascular Imaging*.

[101] Budoff MJ, Shaw LJ, Liu ST, Weinstein SR, Mosler TP, Tseng PH (2007). Long-term prognosis associated with coronary calcification: observations from a registry of 25,253 patients. *Journal of the American College of Cardiology*.

[102] Detrano R, Guerci AD, Carr JJ, Bild DE, Burke G, Folsom AR (2008). Coronary calcium as a predictor of coronary events in four racial or ethnic groups. *The New England Journal of Medicine*.

[103] Schuijf JD, Wijns W, Jukema JW, Decramer I, Atsma DE, de Roos A (2006). A comparative regional analysis of coronary atherosclerosis and calcium score on multislice CT versus myocardial perfusion on SPECT. *Journal of Nuclear Medicine*.

[104] Bavishi C, Argulian E, Chatterjee S, Rozanski A (2016). CACS and the Frequency of Stress-Induced Myocardial Ischemia During MPI: A Meta-Analysis. *JACC: Cardiovascular Imaging*.

[105] Wolk MJ, Bailey SR, Doherty JU, Douglas PS, Hendel RC, Kramer CM (2014). ACCF/AHA/ASE/ASNC/HFSA/HRS/SCAI/SCCT/SCMR/STS 2013 multimodality appropriate use criteria for the detection and risk assessment of stable ischemic heart disease: a report of the American College of Cardiology Foundation Appropriate Use Criteria Task Force, American Heart Association, American Society of Echocardiography, American Society of Nuclear Cardiology, Heart Failure Society of America, Heart Rhythm Society, Society for Cardiovascular Angiography and Interventions, Society of Cardiovascular Computed Tomography, Society for Cardiovascular Magnetic Resonance, and Society of Thoracic Surgeons. *Journal of the American College of Cardiology*.

[106] Patchett ND, Pawar S, Miller EJ (2017). Visual identification of coronary calcifications on attenuation correction CT improves diagnostic accuracy of SPECT/CT myocardial perfusion imaging. *Journal of Nuclear Cardiology*.

[107] Uebleis C, Becker A, Griesshammer I, Cumming P, Becker C, Schmidt M (2009). Stable coronary artery disease: prognostic value of myocardial perfusion SPECT in relation to coronary calcium scoring–long-term follow-up. *Radiology*.

[108] Yokota S, Mouden M, Ottervanger JP, Engbers E, Jager PL, Timmer JR (2019). Coronary calcium score influences referral for invasive coronary angiography after normal myocardial perfusion SPECT. *Journal of Nuclear Cardiology*.

[109] Mouden M, Ottervanger JP, Timmer JR, Reiffers S, Oostdijk AHJ, Knollema S (2014). The influence of coronary calcium score on the interpretation of myocardial perfusion imaging. *Journal of Nuclear Cardiology*.

[110] Anand DV, Lim E, Hopkins D, Corder R, Shaw LJ, Sharp P (2006). Risk stratification in uncomplicated type 2 diabetes: prospective evaluation of the combined use of coronary artery calcium imaging and selective myocardial perfusion scintigraphy. *European Heart Journal*.

[111] Mouden M, Timmer JR, Reiffers S, Oostdijk AHJ, Knollema S, Ottervanger JP (2013). Coronary artery calcium scoring to exclude flow-limiting coronary artery disease in symptomatic stable patients at low or intermediate risk. *Radiology*.

[112] van Werkhoven JM, de Boer SM, Schuijf JD, Cademartiri F, Maffei E, Jukema JW (2010). Impact of clinical presentation and pretest likelihood on the relation between calcium score and computed tomographic coronary angiography. *The American Journal of Cardiology*.

[113] Berman DS, Wong ND, Gransar H, Miranda-Peats R, Dahlbeck J, Hayes SW (2004). Relationship between stress-induced myocardial ischemia and atherosclerosis measured by coronary calcium tomography. *Journal of the American College of Cardiology*.

[114] Ghadri JR, Fiechter M, Fuchs TA, Scherrer A, Stehli J, Gebhard C (2013). Registry for the Evaluation of the PROgnostic value of a novel integrated imaging approach combining Single Photon Emission Computed Tomography with coronary calcification imaging (REPROSPECT). *European Heart Journal - Cardiovascular Imaging*.

[115] Anand DV, Lim E, Raval U, Lipkin D, Lahiri A (2004). Prevalence of silent myocardial ischemia in asymptomatic individuals with subclinical atherosclerosis detected by electron beam tomography. *Journal of Nuclear Cardiology*.

[116] Wong ND, Rozanski A, Gransar H, Miranda-Peats R, Kang X, Hayes S (2005). Metabolic syndrome and diabetes are associated with an increased likelihood of inducible myocardial ischemia among patients with subclinical atherosclerosis. *Diabetes Care*.

[117] von Ziegler F, Brendel M, Uebleis C, Helbig S, Greif M, Ruemmler J (2012). SPECT myocardial perfusion imaging as an adjunct to coronary calcium score for the detection of hemodynamically significant coronary artery stenosis. *BMC Cardiovascular Disorders*.

[118] Schepis T, Gaemperli O, Koepfli P, Namdar M, Valenta I, Scheffel H (2007). Added value of coronary artery calcium score as an adjunct to gated SPECT for the evaluation of coronary artery disease in an intermediate-risk population. *Journal of Nuclear Medicine*.

[119] Miller JM, Rochitte CE, Dewey M, Arbab-Zadeh A, Niinuma H, Gottlieb I (2008). Diagnostic performance of coronary angiography by 64-row CT. *The New England Journal of Medicine*.

[120] Meijboom WB, Meijs MFL, Schuijf JD, Cramer MJ, Mollet NR, van Mieghem CAG (2008). Diagnostic accuracy of 64-slice computed tomography coronary angiography: a prospective, multicenter, multivendor study. *Journal of the American College of Cardiology*.

[121] Javadi MS, Lautamäki R, Merrill J, Voicu C, Epley W, McBride G (2010). Definition of vascular territories on myocardial perfusion images by integration with true coronary anatomy: a hybrid PET/CT analysis. *Journal of Nuclear Medicine*.

[122] Koo B, Erglis A, Doh J, Daniels DV, Jegere S, Kim H (2011). Diagnosis of ischemia-causing coronary stenoses by noninvasive fractional flow reserve computed from coronary computed tomographic angiograms. Results from the prospective multicenter DISCOVER-FLOW (Diagnosis of Ischemia-Causing Stenoses Obtained Via Noninvasive Fractional Flow Reserve) study. *Journal of the American College of Cardiology*.

[123] Di Carli M, Czernin J, Hoh CK, Gerbaudo VH, Brunken RC, Huang SC (1995). Relation among stenosis severity, myocardial blood flow, and flow reserve in patients with coronary artery disease. *Circulation*.

[124] Neglia D, Liga R, Caselli C, Carpeggiani C, Lorenzoni V, Sicari R (2020). Anatomical and functional coronary imaging to predict long-term outcome in patients with suspected coronary artery disease: the EVINCI-outcome study. *European Heart Journal - Cardiovascular Imaging*.

[125] Schaap J, Kauling RM, Boekholdt SM, Nieman K, Meijboom WB, Post MC (2013). Incremental diagnostic accuracy of hybrid SPECT/CT coronary angiography in a population with an intermediate to high pre-test likelihood of coronary artery disease. *European Heart Journal. Cardiovascular Imaging*.

[126] Seraphim A, Knott KD, Augusto JB, Menacho K, Tyebally S, Dowsing B (2021). Non-invasive Ischaemia Testing in Patients With Prior Coronary Artery Bypass Graft Surgery: Technical Challenges, Limitations, and Future Directions. *Frontiers in Cardiovascular Medicine*.

[127] Maaniitty T, Jaakkola S, Saraste A, Knuuti J (2019). Hybrid coronary computed tomography angiography and positron emission tomography myocardial perfusion imaging in evaluation of recurrent symptoms after coronary artery bypass grafting. *European Heart Journal - Cardiovascular Imaging*.

[128] Kawai H, Sarai M, Motoyama S, Ito H, Takada K, Harigaya H (2013). A combination of anatomical and functional evaluations improves the prediction of cardiac event in patients with coronary artery bypass. *BMJ Open*.

[129] Kim H, Kim Y, Lee S, Park E, Paeng J, Kim H (2014). Incremental prognostic value of sequential imaging of single-photon emission computed tomography and coronary computed tomography angiography in patients with suspected coronary artery disease. *European Heart Journal - Cardiovascular Imaging*.

[130] Hoffmann U, Ferencik M, Udelson JE, Picard MH, Truong QA, Patel MR (2017). Prognostic Value of Noninvasive Cardiovascular Testing in Patients With Stable Chest Pain: Insights From the PROMISE Trial (Prospective Multicenter Imaging Study for Evaluation of Chest Pain). *Circulation*.

[131] Min JK, Dunning A, Lin FY, Achenbach S, Al-Mallah M, Budoff MJ (2011). Age- and sex-related differences in all-cause mortality risk based on coronary computed tomography angiography findings results from the International Multicenter CONFIRM (Coronary CT Angiography Evaluation for Clinical Outcomes: An International Multicenter Registry) of 23,854 patients without known coronary artery disease. *Journal of the American College of Cardiology*.

[132] Lee H, Yoon YE, Lee W, Choi H, Park J, Kim H (2018). Prognosis of anatomic coronary artery disease without myocardial ischemia: Coronary computed tomography angiography detects high-risk patients even in cases of negative single-photon emission computed tomography findings. *Journal of Cardiology*.

[133] Slomka PJ, Cheng VY, Dey D, Woo J, Ramesh A, Van Kriekinge S (2009). Quantitative analysis of myocardial perfusion SPECT anatomically guided by coregistered 64-slice coronary CT angiography. *Journal of Nuclear Medicine*.

[134] Takx RAP, Blomberg BA, El Aidi H, Habets J, de Jong PA, Nagel E (2015). Diagnostic accuracy of stress myocardial perfusion imaging compared to invasive coronary angiography with fractional flow reserve meta-analysis. *Circulation: Cardiovascular Imaging*.

[135] George RT, Mehra VC, Chen MY, Kitagawa K, Arbab-Zadeh A, Miller JM (2014). Myocardial CT perfusion imaging and SPECT for the diagnosis of coronary artery disease: a head-to-head comparison from the CORE320 multicenter diagnostic performance study. *Radiology*.

[136] Yang DH, Kim Y (2017). CT myocardial perfusion imaging: current status and future perspectives. *The International Journal of Cardiovascular Imaging*.

[137] Pijls NHJ, Fearon WF, Tonino PAL, Siebert U, Ikeno F, Bornschein B (2010). Fractional flow reserve versus angiography for guiding percutaneous coronary intervention in patients with multivessel coronary artery disease: 2-year follow-up of the FAME (Fractional Flow Reserve Versus Angiography for Multivessel Evaluation) study. *Journal of the American College of Cardiology*.

[138] Pijls NHJ, van Schaardenburgh P, Manoharan G, Boersma E, Bech J, van’t Veer M (2007). Percutaneous coronary intervention of functionally nonsignificant stenosis: 5-year follow-up of the DEFER Study. *Journal of the American College of Cardiology*.

[139] Koo HJ, Yang DH, Kim Y, Kang J, Kang S, Kweon J (2016). CT-based myocardial ischemia evaluation: quantitative angiography, transluminal attenuation gradient, myocardial perfusion, and CT-derived fractional flow reserve. *The International Journal of Cardiovascular Imaging*.

[140] Gimelli A, Liga R (2019). Comparative accuracy of myocardial perfusion imaging: The final answer has yet to come. *International Journal of Cardiology*.

